# Crystal structure of propaquizafop

**DOI:** 10.1107/S1600536814024751

**Published:** 2014-11-19

**Authors:** Youngeun Jeon, Jineun Kim, Sangjin Lee, Tae Ho Kim

**Affiliations:** a Department of Chemistry and Research Institute of Natural Sciences, Gyeongsang National University, Jinju 660-701, Republic of Korea

**Keywords:** crystal structure, propaquizafop, herbicide, hydrogen bonding, π–π inter­actions

## Abstract

The title compound, C_22_H_22_ClN_3_O_5_ {systematic name: 2-(propan-2-yl­idene­amino­oxy)ethyl (*R*)-2-[4-(6-chloro­quin­oxalin-2-yl­oxy)phen­oxy]propionate}, is a herbicide. The asymmetric unit comprises two independent mol­ecules in which the dihedral angles between the phenyl ring and the quinoxaline ring plane are 75.93 (7) and 82.77 (8)°. The crystal structure features C—H⋯O, C—H⋯N, and C—H⋯Cl hydrogen bonds, as well as weak π–π inter­actions [ring-centroid separation = 3.782 (2) and 3.5952 (19) Å], resulting in a three-dimensional architecture.

## Related literature   

For information on the herbicidal properties of the title compound, see: Bergkvist & Ledin (1997 ▶). For a related crystal structure, see: Hu *et al.* (2009 ▶).
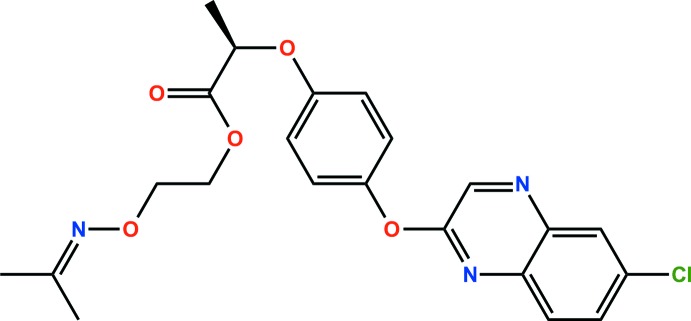


## Experimental   

### Crystal data  

C_22_H_22_ClN_3_O_5_
*M* *_r_* = 443.88
Monoclinic, 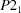

*a* = 4.6205 (2) Å
*b* = 21.9423 (10) Å
*c* = 21.1737 (10) Å
β = 93.263 (2)°
*V* = 2143.20 (17) Å^3^
*Z* = 4
Mo *K*α radiation
μ = 0.22 mm^−1^
*T* = 173 K
0.47 × 0.05 × 0.04 mm

### Data collection  

Bruker APEXII CCD diffractometer
Absorption correction: multi-scan (*SADABS*; Bruker, 2009 ▶) *T*_min_ = 0.905, *T*_max_ = 0.991
20815 measured reflections
8319 independent reflections
5996 reflections with *I* > 2σ(*I*)
*R*_int_ = 0.063

### Refinement  

*R*[*F* ^2^ > 2σ(*F* ^2^)] = 0.054
*wR*(*F* ^2^) = 0.103
*S* = 0.99
8319 reflections
565 parameters
1 restraint
H-atom parameters constrained
Δρ_max_ = 0.26 e Å^−3^
Δρ_min_ = −0.24 e Å^−3^
Absolute structure: Flack (1983 ▶), 4004 Friedel pairs
Absolute structure parameter: 0.05 (6)

### 

Data collection: *APEX2* (Bruker, 2009 ▶); cell refinement: *SAINT* (Bruker, 2009 ▶); data reduction: *SAINT*; program(s) used to solve structure: *SHELXTL* (Sheldrick, 2008 ▶); program(s) used to refine structure: *SHELXTL*; molecular graphics: *DIAMOND* (Brandenburg, 2010 ▶); software used to prepare material for publication: *SHELXTL*.

## Supplementary Material

Crystal structure: contains datablock(s) global, I. DOI: 10.1107/S1600536814024751/hg5418sup1.cif

Structure factors: contains datablock(s) I. DOI: 10.1107/S1600536814024751/hg5418Isup2.hkl

Supporting information file. DOI: 10.1107/S1600536814024751/hg5418Isup3.cml

**Fig. 1.**
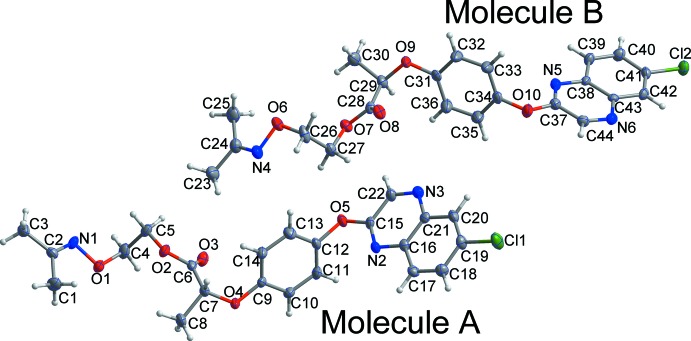
The asymmetric unit of the title compound with the atom numbering scheme. Displacement ellipsoids are drawn at the 50% probability level. H atoms are shown as small spheres of arbitrary radius.

**Fig. 2.**
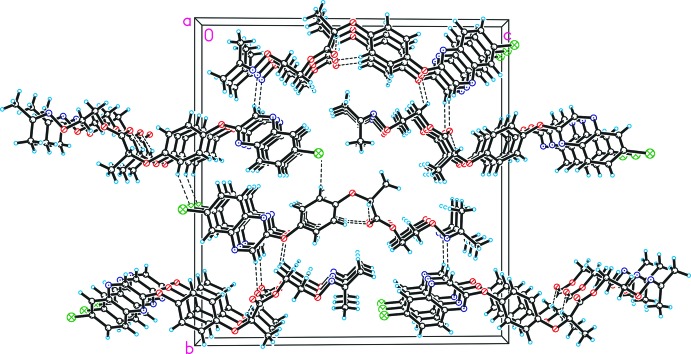
Crystal packing viewed along the a axis. The intermolecular C—H···N, C—H···O, and C—H···Cl hydrogen bonds are shown as dashed lines.

. DOI: 10.1107/S1600536814024751/hg5418fig1.tif
The asymmetric unit of the title compound with the atom numbering scheme. Displacement ellipsoids are drawn at the 50% probability level. H atoms are shown as small spheres of arbitrary radius.

a . DOI: 10.1107/S1600536814024751/hg5418fig2.tif
Crystal packing viewed along the *a* axis. The inter­molecular C—H⋯N, C—H⋯O, and C—H⋯Cl hydrogen bonds are shown as dashed lines.

CCDC reference: 1033575

Additional supporting information:  crystallographic information; 3D view; checkCIF report

## Figures and Tables

**Table 1:** Hydrogen-bond geometry (, )

*D*H*A*	*D*H	H*A*	*D* *A*	*D*H*A*
C4H4*B*O2^i^	0.99	2.60	3.337(4)	131
C7H7O3^ii^	1.00	2.33	3.264(4)	154
C14H14O3^ii^	0.95	2.47	3.389(4)	164
C22H22O8^i^	0.95	2.53	3.468(4)	170
C27H27*A*O5^ii^	0.99	2.56	3.284(4)	130
C29H29O8^i^	1.00	2.33	3.173(4)	142
C44H44N1^iii^	0.95	2.51	3.430(5)	162
C10H10Cl2^iv^	0.95	2.91	3.855(4)	174
C25H25*A*Cl2^v^	0.98	2.99	3.368(4)	105
C32H32Cl1^vi^	0.95	2.89	3.748(4)	151

Symmetry codes: (i) 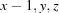; (ii) 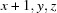; (iii) 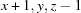; (iv) 
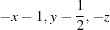
; (v) 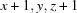; (vi) 
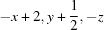
.

## supplementary crystallographic information

### S1. Comment

Propaquizafop, C_22_H_22_ClN_3_O_5_, is a herbicide of the aryloxy phenoxy propionate family. It is used for the post emergence control of a wide range of annual and perennial grasses. It is used for selective weed control in many broadleaf crops such as sugarbeet, oilseed rape, soybeans, sunflower, other field crops, vegetables, fruit trees, vineyards and forestry. (Bergkvist & Ledin, 1997). Its crystal structure is reported herein. In this compound (Scheme 1, Fig. 1), there are two independent and conformationally similar molecules (*A* and *B*) in the asymmetric unit, with the dihedral angle between the phenyl ring and quinoxaline ring planes are 75.93 (7) and 82.77 (8)°. All bond lengths and bond angles are normal and comparable to those observed in the crystal structure of a similar compound (Hu *et al.*, 2009).

In the crystal structure (Fig. 2), The crystal structure is stabilized by C—H···O, C—H···N, and C—H···Cl hydrogen bonds (Table 1), as well as two weak intermolecular π–π interactions between the quinoxaline rings [*Cg*1···*Cg*2^i^ = 3.782 (2) Å. *Cg*1 and *Cg*2 are the centroids of the C15—N2—C16—C21—N3—C22 and the C16—C21 rings, respectively. *Cg*3···*Cg*4^ii^ = 3.5952 (19) Å. *Cg*3 and *Cg*4 are the centroid of the C37—N5—C38—C43—N6—C44 and the C38—C43 rings, respectively. (Symmetry codes: (i), *x* - 1, *y*, *z* and (ii), *x* + 1, *y*, *z*)], resulting in a three-dimensional architecture.

### S2. Experimental

The title compound was purchased from the Dr. Ehrenstorfer GmbH Company. Slow evaporation of a solution in CH_3_OH gave single crystals suitable for X-ray analysis.

### S3. Refinement

All H-atoms were positioned geometrically and refined using a riding model with d(C—H) = 0.98 Å, *U*_iso_ = 1.5*U*_eq_(C) for methyl group, d(C—H) = 0.99 Å, *U*_iso_ = 1.2*U*_eq_(C) for C*sp*^3^—H, d(C—H) = 0.95 Å, *U*_iso_ = 1.2*U*_eq_(C) for aromatic C—H, and d(C—H) = 1.00 Å, *U*_iso_ = 1.5*U*_eq_(C) for C*sp*^3^—H.

### Crystal data

**Table:**

C_22_H_22_ClN_3_O_5_	*F*(000) = 928
*M**_r_* = 443.88	*D*_x_ = 1.376 Mg m^−^^3^
Monoclinic, *P*2_1_	Mo *K*α radiation, λ = 0.71073 Å
Hall symbol: P 2yb	Cell parameters from 2323 reflections
*a* = 4.6205 (2) Å	θ = 2.7–19.3°
*b* = 21.9423 (10) Å	µ = 0.22 mm^−^^1^
*c* = 21.1737 (10) Å	*T* = 173 K
β = 93.263 (2)°	Needle, colourless
*V* = 2143.20 (17) Å^3^	0.47 × 0.05 × 0.04 mm
*Z* = 4	

### Data collection

**Table:**

Bruker APEXII CCD diffractometer	8319 independent reflections
Radiation source: fine-focus sealed tube	5996 reflections with *I* > 2σ(*I*)
Graphite monochromator	*R*_int_ = 0.063
φ and ω scans	θ_max_ = 26.0°, θ_min_ = 1.9°
Absorption correction: multi-scan (*SADABS*; Bruker, 2009)	*h* = −5→5
*T*_min_ = 0.905, *T*_max_ = 0.991	*k* = −26→27
20815 measured reflections	*l* = −26→26

### Refinement

**Table:**

Refinement on *F*^2^	Secondary atom site location: difference Fourier map
Least-squares matrix: full	Hydrogen site location: inferred from neighbouring sites
*R*[*F*^2^ > 2σ(*F*^2^)] = 0.054	H-atom parameters constrained
*wR*(*F*^2^) = 0.103	*w* = 1/[σ^2^(*F*_o_^2^) + (0.0354*P*)^2^] where *P* = (*F*_o_^2^ + 2*F*_c_^2^)/3
*S* = 0.99	(Δ/σ)_max_ < 0.001
8319 reflections	Δρ_max_ = 0.26 e Å^−^^3^
565 parameters	Δρ_min_ = −0.24 e Å^−^^3^
1 restraint	Absolute structure: Flack (1983), 4004 Friedel pairs
Primary atom site location: structure-invariant direct methods	Absolute structure parameter: 0.05 (6)

### Special details

**Table:**

Geometry. All e.s.d.'s (except the e.s.d. in the dihedral angle between two l.s. planes) are estimated using the full covariance matrix. The cell e.s.d.'s are taken into account individually in the estimation of e.s.d.'s in distances, angles and torsion angles; correlations between e.s.d.'s in cell parameters are only used when they are defined by crystal symmetry. An approximate (isotropic) treatment of cell e.s.d.'s is used for estimating e.s.d.'s involving l.s. planes.
Refinement. Refinement of *F*^2^ against ALL reflections. The weighted *R*-factor *wR* and goodness of fit *S* are based on *F*^2^, conventional *R*-factors *R* are based on *F*, with *F* set to zero for negative *F*^2^. The threshold expression of *F*^2^ > σ(*F*^2^) is used only for calculating *R*-factors(gt) *etc*. and is not relevant to the choice of reflections for refinement. *R*-factors based on *F*^2^ are statistically about twice as large as those based on *F*, and *R*- factors based on ALL data will be even larger.

### Fractional atomic coordinates and isotropic or equivalent isotropic displacement parameters (Å^2^)

**Table:**

	*x*	*y*	*z*	*U*_iso_*/*U*_eq_	
Cl1	0.8646 (2)	0.58056 (5)	−0.01482 (4)	0.0403 (3)	
Cl2	−0.2992 (2)	0.91749 (4)	−0.39805 (4)	0.0328 (2)	
O1	−0.0906 (5)	0.62880 (10)	0.75631 (10)	0.0300 (6)	
O2	0.1079 (5)	0.64050 (10)	0.62762 (10)	0.0263 (6)	
O3	−0.2656 (5)	0.63233 (11)	0.55516 (10)	0.0296 (6)	
O4	−0.0116 (6)	0.53477 (11)	0.49904 (10)	0.0282 (6)	
O5	−0.0140 (6)	0.67388 (11)	0.28083 (10)	0.0305 (6)	
O6	0.6212 (6)	0.84357 (11)	0.39165 (11)	0.0323 (6)	
O7	0.3833 (5)	0.83763 (10)	0.25968 (10)	0.0254 (6)	
O8	0.7468 (5)	0.84570 (11)	0.19415 (10)	0.0291 (6)	
O9	0.4940 (5)	0.94441 (10)	0.13532 (10)	0.0281 (6)	
O10	0.6648 (5)	0.82773 (10)	−0.09140 (10)	0.0315 (6)	
N1	−0.0042 (7)	0.67417 (13)	0.80229 (13)	0.0281 (7)	
N2	0.2541 (6)	0.60906 (13)	0.22060 (12)	0.0235 (7)	
N3	0.2624 (7)	0.70563 (13)	0.13081 (13)	0.0286 (7)	
N4	0.5470 (7)	0.79627 (13)	0.43496 (13)	0.0328 (8)	
N5	0.3225 (6)	0.88493 (13)	−0.14960 (12)	0.0241 (7)	
N6	0.4407 (7)	0.80739 (13)	−0.25283 (14)	0.0264 (7)	
C1	0.2912 (9)	0.58894 (17)	0.84480 (18)	0.0407 (10)	
H1A	0.1313	0.5597	0.8463	0.061*	
H1B	0.4244	0.5834	0.8821	0.061*	
H1C	0.3954	0.5821	0.8064	0.061*	
C2	0.1739 (8)	0.65195 (16)	0.84430 (16)	0.0260 (8)	
C3	0.2718 (9)	0.69528 (17)	0.89626 (16)	0.0369 (10)	
H3A	0.1748	0.7346	0.8894	0.055*	
H3B	0.4821	0.7009	0.8960	0.055*	
H3C	0.2222	0.6785	0.9372	0.055*	
C4	−0.2490 (8)	0.65898 (18)	0.70522 (16)	0.0330 (9)	
H4A	−0.3731	0.6910	0.7226	0.040*	
H4B	−0.3770	0.6292	0.6823	0.040*	
C5	−0.0510 (8)	0.68746 (16)	0.65971 (16)	0.0288 (9)	
H5A	−0.1664	0.7120	0.6281	0.035*	
H5B	0.0873	0.7150	0.6829	0.035*	
C6	−0.0370 (8)	0.61396 (16)	0.57733 (15)	0.0231 (8)	
C7	0.1219 (8)	0.55835 (15)	0.55631 (15)	0.0239 (8)	
H7	0.3296	0.5684	0.5504	0.029*	
C8	0.1005 (10)	0.50749 (17)	0.60594 (16)	0.0375 (10)	
H8A	−0.1038	0.4994	0.6129	0.056*	
H8B	0.2005	0.5203	0.6458	0.056*	
H8C	0.1912	0.4703	0.5907	0.056*	
C9	0.0115 (8)	0.56995 (15)	0.44475 (15)	0.0220 (8)	
C10	−0.1660 (8)	0.55205 (16)	0.39339 (16)	0.0293 (9)	
H10	−0.2871	0.5173	0.3964	0.035*	
C11	−0.1670 (8)	0.58495 (17)	0.33742 (16)	0.0291 (9)	
H11	−0.2895	0.5732	0.3019	0.035*	
C12	0.0091 (8)	0.63397 (16)	0.33417 (15)	0.0253 (9)	
C13	0.1930 (8)	0.65152 (18)	0.38381 (16)	0.0332 (9)	
H13	0.3166	0.6858	0.3799	0.040*	
C14	0.1968 (8)	0.61839 (16)	0.44033 (16)	0.0278 (9)	
H14	0.3257	0.6293	0.4751	0.033*	
C15	0.1284 (8)	0.66053 (16)	0.22798 (15)	0.0234 (8)	
C16	0.3954 (7)	0.60351 (15)	0.16469 (15)	0.0219 (8)	
C17	0.5290 (8)	0.54843 (15)	0.15090 (16)	0.0252 (8)	
H17	0.5228	0.5155	0.1800	0.030*	
C18	0.6692 (8)	0.54103 (17)	0.09599 (16)	0.0289 (9)	
H18	0.7572	0.5032	0.0866	0.035*	
C19	0.6801 (8)	0.59032 (17)	0.05409 (15)	0.0279 (9)	
C20	0.5540 (8)	0.64459 (17)	0.06578 (16)	0.0282 (9)	
H20	0.5678	0.6775	0.0369	0.034*	
C21	0.4019 (7)	0.65187 (16)	0.12123 (15)	0.0213 (8)	
C22	0.1234 (8)	0.70974 (17)	0.18315 (15)	0.0289 (9)	
H22	0.0181	0.7458	0.1915	0.035*	
C23	0.2920 (10)	0.77216 (18)	0.52389 (18)	0.0457 (12)	
H23A	0.3871	0.7331	0.5164	0.069*	
H23B	0.0812	0.7666	0.5207	0.069*	
H23C	0.3522	0.7871	0.5662	0.069*	
C24	0.3764 (8)	0.81741 (17)	0.47554 (17)	0.0305 (9)	
C25	0.2581 (9)	0.88020 (18)	0.47602 (18)	0.0398 (10)	
H25A	0.4160	0.9095	0.4721	0.060*	
H25B	0.1652	0.8873	0.5158	0.060*	
H25C	0.1149	0.8853	0.4405	0.060*	
C26	0.7593 (8)	0.81513 (19)	0.34125 (16)	0.0341 (10)	
H26A	0.8859	0.7819	0.3583	0.041*	
H26B	0.8828	0.8453	0.3207	0.041*	
C27	0.5417 (8)	0.78956 (17)	0.29305 (16)	0.0278 (9)	
H27A	0.6428	0.7643	0.2624	0.033*	
H27B	0.4042	0.7630	0.3144	0.033*	
C28	0.5173 (7)	0.86318 (16)	0.21119 (15)	0.0212 (8)	
C29	0.3410 (7)	0.91649 (16)	0.18402 (14)	0.0255 (8)	
H29	0.1471	0.9020	0.1668	0.031*	
C30	0.3044 (9)	0.96437 (17)	0.23452 (17)	0.0403 (10)	
H30A	0.4955	0.9787	0.2506	0.060*	
H30B	0.2015	0.9466	0.2693	0.060*	
H30C	0.1926	0.9987	0.2163	0.060*	
C31	0.5158 (8)	0.91335 (16)	0.07887 (15)	0.0246 (8)	
C32	0.6921 (9)	0.93993 (17)	0.03696 (16)	0.0409 (11)	
H32	0.7851	0.9774	0.0477	0.049*	
C33	0.7369 (9)	0.91307 (18)	−0.02070 (16)	0.0377 (10)	
H33	0.8607	0.9316	−0.0495	0.045*	
C34	0.6009 (8)	0.85980 (16)	−0.03539 (15)	0.0268 (9)	
C35	0.4212 (9)	0.83233 (18)	0.00528 (17)	0.0390 (10)	
H35	0.3267	0.7952	−0.0062	0.047*	
C36	0.3778 (9)	0.85927 (18)	0.06367 (17)	0.0386 (10)	
H36	0.2550	0.8406	0.0925	0.046*	
C37	0.5101 (8)	0.84137 (16)	−0.14593 (15)	0.0243 (8)	
C38	0.1798 (7)	0.89193 (15)	−0.20807 (15)	0.0200 (8)	
C39	−0.0240 (8)	0.93925 (15)	−0.21804 (15)	0.0261 (9)	
H39	−0.0634	0.9656	−0.1840	0.031*	
C40	−0.1663 (8)	0.94788 (16)	−0.27590 (16)	0.0279 (9)	
H40	−0.2995	0.9806	−0.2825	0.034*	
C41	−0.1115 (7)	0.90744 (16)	−0.32525 (14)	0.0240 (8)	
C42	0.0835 (8)	0.86119 (15)	−0.31783 (15)	0.0238 (8)	
H42	0.1156	0.8344	−0.3520	0.029*	
C43	0.2374 (7)	0.85328 (14)	−0.25905 (15)	0.0201 (8)	
C44	0.5739 (8)	0.80277 (16)	−0.19749 (16)	0.0259 (9)	
H44	0.7195	0.7725	−0.1910	0.031*	

### Atomic displacement parameters (Å^2^)

**Table:**

	*U*^11^	*U*^22^	*U*^33^	*U*^12^	*U*^13^	*U*^23^
Cl1	0.0453 (6)	0.0483 (6)	0.0288 (5)	0.0108 (5)	0.0144 (5)	0.0064 (5)
Cl2	0.0361 (5)	0.0393 (6)	0.0225 (5)	0.0037 (5)	−0.0025 (4)	−0.0023 (4)
O1	0.0389 (16)	0.0270 (14)	0.0235 (13)	0.0008 (12)	−0.0035 (12)	−0.0057 (11)
O2	0.0294 (14)	0.0267 (14)	0.0223 (13)	0.0021 (12)	−0.0034 (11)	−0.0083 (11)
O3	0.0254 (15)	0.0379 (16)	0.0249 (13)	0.0003 (13)	−0.0017 (12)	−0.0050 (12)
O4	0.0424 (16)	0.0261 (14)	0.0157 (12)	−0.0112 (12)	−0.0006 (11)	−0.0004 (11)
O5	0.0466 (17)	0.0275 (14)	0.0178 (13)	0.0077 (12)	0.0063 (12)	0.0043 (11)
O6	0.0375 (16)	0.0358 (15)	0.0241 (13)	−0.0003 (13)	0.0052 (12)	0.0088 (12)
O7	0.0231 (13)	0.0284 (14)	0.0250 (13)	0.0026 (12)	0.0038 (11)	0.0060 (11)
O8	0.0222 (14)	0.0377 (15)	0.0276 (14)	0.0018 (12)	0.0035 (12)	0.0045 (12)
O9	0.0397 (16)	0.0276 (14)	0.0170 (12)	−0.0090 (12)	0.0032 (11)	0.0008 (11)
O10	0.0419 (16)	0.0302 (15)	0.0216 (13)	0.0092 (13)	−0.0050 (12)	−0.0034 (12)
N1	0.0331 (19)	0.0289 (17)	0.0220 (16)	−0.0028 (15)	−0.0009 (14)	−0.0100 (14)
N2	0.0311 (18)	0.0234 (16)	0.0158 (15)	0.0000 (14)	−0.0011 (13)	0.0029 (13)
N3	0.0353 (19)	0.0267 (17)	0.0235 (17)	−0.0002 (15)	−0.0017 (15)	0.0017 (14)
N4	0.043 (2)	0.0327 (19)	0.0233 (16)	−0.0008 (16)	0.0065 (15)	0.0046 (14)
N5	0.0247 (17)	0.0284 (17)	0.0193 (16)	−0.0030 (14)	0.0013 (13)	0.0013 (13)
N6	0.0309 (18)	0.0244 (17)	0.0243 (16)	0.0005 (14)	0.0055 (14)	−0.0015 (13)
C1	0.053 (3)	0.030 (2)	0.038 (2)	0.012 (2)	−0.011 (2)	0.0010 (19)
C2	0.028 (2)	0.026 (2)	0.0237 (19)	−0.0005 (18)	0.0001 (17)	0.0068 (17)
C3	0.048 (3)	0.034 (2)	0.027 (2)	−0.013 (2)	−0.0103 (19)	0.0029 (18)
C4	0.034 (2)	0.040 (2)	0.024 (2)	0.0076 (19)	−0.0062 (18)	−0.0059 (18)
C5	0.035 (2)	0.027 (2)	0.024 (2)	0.0032 (18)	−0.0065 (18)	−0.0080 (17)
C6	0.029 (2)	0.025 (2)	0.0155 (18)	−0.0086 (17)	0.0015 (17)	0.0016 (15)
C7	0.032 (2)	0.022 (2)	0.0173 (18)	−0.0014 (17)	−0.0016 (16)	0.0011 (15)
C8	0.059 (3)	0.033 (2)	0.020 (2)	−0.002 (2)	−0.0043 (19)	0.0050 (17)
C9	0.032 (2)	0.0193 (19)	0.0152 (17)	0.0006 (17)	0.0040 (16)	−0.0033 (15)
C10	0.042 (3)	0.022 (2)	0.023 (2)	−0.0073 (18)	−0.0009 (18)	−0.0033 (16)
C11	0.038 (2)	0.030 (2)	0.0189 (19)	−0.0020 (19)	−0.0052 (17)	−0.0034 (17)
C12	0.037 (2)	0.024 (2)	0.0153 (18)	0.0048 (18)	0.0052 (17)	0.0036 (16)
C13	0.038 (2)	0.034 (2)	0.028 (2)	−0.008 (2)	0.0040 (18)	0.0044 (18)
C14	0.034 (2)	0.030 (2)	0.0196 (19)	−0.0085 (18)	0.0007 (17)	−0.0008 (16)
C15	0.029 (2)	0.027 (2)	0.0139 (18)	−0.0001 (17)	−0.0020 (16)	−0.0030 (16)
C16	0.0194 (19)	0.027 (2)	0.0194 (18)	0.0013 (16)	0.0000 (15)	0.0009 (16)
C17	0.026 (2)	0.025 (2)	0.0254 (19)	−0.0038 (17)	0.0040 (17)	0.0040 (16)
C18	0.027 (2)	0.029 (2)	0.030 (2)	0.0049 (18)	0.0011 (17)	0.0008 (18)
C19	0.026 (2)	0.036 (2)	0.0222 (19)	0.0042 (18)	0.0059 (16)	0.0016 (18)
C20	0.031 (2)	0.034 (2)	0.0192 (19)	−0.0055 (19)	0.0001 (16)	0.0060 (17)
C21	0.0196 (19)	0.0236 (19)	0.0201 (18)	−0.0028 (16)	−0.0040 (15)	−0.0018 (16)
C22	0.039 (2)	0.029 (2)	0.0194 (19)	0.0053 (18)	0.0025 (18)	0.0009 (17)
C23	0.068 (3)	0.037 (2)	0.033 (2)	−0.003 (2)	0.016 (2)	0.003 (2)
C24	0.031 (2)	0.036 (2)	0.024 (2)	0.0034 (19)	0.0017 (18)	−0.0006 (18)
C25	0.040 (3)	0.045 (3)	0.033 (2)	0.008 (2)	−0.0057 (19)	0.002 (2)
C26	0.028 (2)	0.054 (3)	0.0202 (19)	0.005 (2)	0.0053 (18)	0.0095 (19)
C27	0.031 (2)	0.031 (2)	0.0220 (19)	0.0077 (18)	0.0042 (17)	0.0046 (17)
C28	0.017 (2)	0.030 (2)	0.0165 (18)	−0.0079 (17)	−0.0005 (16)	−0.0017 (16)
C29	0.027 (2)	0.029 (2)	0.0208 (18)	−0.0007 (18)	0.0049 (15)	0.0035 (17)
C30	0.057 (3)	0.031 (2)	0.034 (2)	0.009 (2)	0.017 (2)	0.0004 (19)
C31	0.031 (2)	0.0233 (19)	0.0184 (18)	0.0026 (18)	−0.0036 (16)	0.0040 (17)
C32	0.064 (3)	0.030 (2)	0.029 (2)	−0.021 (2)	0.014 (2)	−0.0051 (19)
C33	0.058 (3)	0.028 (2)	0.029 (2)	−0.010 (2)	0.015 (2)	−0.0014 (19)
C34	0.034 (2)	0.028 (2)	0.0175 (19)	0.0055 (18)	−0.0023 (17)	0.0023 (16)
C35	0.053 (3)	0.035 (2)	0.028 (2)	−0.018 (2)	−0.002 (2)	−0.0017 (19)
C36	0.047 (3)	0.044 (3)	0.024 (2)	−0.018 (2)	0.0026 (19)	−0.0025 (19)
C37	0.029 (2)	0.023 (2)	0.0212 (19)	−0.0014 (18)	0.0015 (16)	0.0007 (16)
C38	0.026 (2)	0.0174 (17)	0.0166 (18)	−0.0062 (16)	0.0021 (15)	−0.0030 (15)
C39	0.030 (2)	0.026 (2)	0.0219 (19)	0.0020 (17)	0.0033 (17)	−0.0062 (16)
C40	0.027 (2)	0.028 (2)	0.029 (2)	0.0051 (17)	0.0014 (17)	−0.0040 (17)
C41	0.0268 (19)	0.030 (2)	0.0154 (17)	−0.0042 (18)	−0.0004 (15)	0.0016 (16)
C42	0.028 (2)	0.0245 (19)	0.0199 (19)	−0.0019 (17)	0.0064 (16)	−0.0062 (16)
C43	0.024 (2)	0.0132 (17)	0.0236 (19)	−0.0045 (15)	0.0032 (16)	0.0005 (15)
C44	0.029 (2)	0.022 (2)	0.027 (2)	0.0024 (17)	0.0046 (18)	0.0017 (16)

### Geometric parameters (Å, º)

**Table:**

Cl1—C19	1.745 (3)	C12—C13	1.369 (5)
Cl2—C41	1.739 (3)	C13—C14	1.399 (5)
O1—C4	1.433 (4)	C13—H13	0.9500
O1—N1	1.433 (3)	C14—H14	0.9500
O2—C6	1.357 (4)	C15—C22	1.437 (5)
O2—C5	1.456 (4)	C16—C17	1.396 (4)
O3—C6	1.201 (4)	C16—C21	1.406 (4)
O4—C9	1.394 (4)	C17—C18	1.372 (5)
O4—C7	1.426 (4)	C17—H17	0.9500
O5—C15	1.362 (4)	C18—C19	1.402 (5)
O5—C12	1.428 (4)	C18—H18	0.9500
O6—C26	1.419 (4)	C19—C20	1.355 (5)
O6—N4	1.439 (3)	C20—C21	1.411 (5)
O7—C28	1.351 (4)	C20—H20	0.9500
O7—C27	1.445 (4)	C22—H22	0.9500
O8—C28	1.202 (4)	C23—C24	1.494 (5)
O9—C31	1.384 (4)	C23—H23A	0.9800
O9—C29	1.422 (4)	C23—H23B	0.9800
O10—C37	1.356 (4)	C23—H23C	0.9800
O10—C34	1.424 (4)	C24—C25	1.482 (5)
N1—C2	1.274 (4)	C25—H25A	0.9800
N2—C15	1.283 (4)	C25—H25B	0.9800
N2—C16	1.390 (4)	C25—H25C	0.9800
N3—C22	1.315 (4)	C26—C27	1.500 (5)
N3—C21	1.365 (4)	C26—H26A	0.9900
N4—C24	1.285 (4)	C26—H26B	0.9900
N5—C37	1.290 (4)	C27—H27A	0.9900
N5—C38	1.378 (4)	C27—H27B	0.9900
N6—C44	1.296 (4)	C28—C29	1.520 (5)
N6—C43	1.378 (4)	C29—C30	1.515 (5)
C1—C2	1.485 (5)	C29—H29	1.0000
C1—H1A	0.9800	C30—H30A	0.9800
C1—H1B	0.9800	C30—H30B	0.9800
C1—H1C	0.9800	C30—H30C	0.9800
C2—C3	1.504 (5)	C31—C32	1.369 (5)
C3—H3A	0.9800	C31—C36	1.376 (5)
C3—H3B	0.9800	C32—C33	1.382 (5)
C3—H3C	0.9800	C32—H32	0.9500
C4—C5	1.502 (5)	C33—C34	1.355 (5)
C4—H4A	0.9900	C33—H33	0.9500
C4—H4B	0.9900	C34—C35	1.370 (5)
C5—H5A	0.9900	C35—C36	1.395 (5)
C5—H5B	0.9900	C35—H35	0.9500
C6—C7	1.504 (5)	C36—H36	0.9500
C7—C8	1.540 (4)	C37—C44	1.426 (5)
C7—H7	1.0000	C38—C39	1.410 (5)
C8—H8A	0.9800	C38—C43	1.410 (4)
C8—H8B	0.9800	C39—C40	1.370 (5)
C8—H8C	0.9800	C39—H39	0.9500
C9—C14	1.371 (5)	C40—C41	1.405 (5)
C9—C10	1.381 (5)	C40—H40	0.9500
C10—C11	1.387 (5)	C41—C42	1.360 (5)
C10—H10	0.9500	C42—C43	1.409 (5)
C11—C12	1.353 (5)	C42—H42	0.9500
C11—H11	0.9500	C44—H44	0.9500
			
C4—O1—N1	107.6 (3)	C21—C20—H20	120.3
C6—O2—C5	115.5 (3)	N3—C21—C16	121.8 (3)
C9—O4—C7	116.8 (2)	N3—C21—C20	118.9 (3)
C15—O5—C12	120.0 (3)	C16—C21—C20	119.3 (3)
C26—O6—N4	107.2 (3)	N3—C22—C15	121.0 (3)
C28—O7—C27	115.7 (3)	N3—C22—H22	119.5
C31—O9—C29	118.5 (3)	C15—C22—H22	119.5
C37—O10—C34	118.4 (3)	C24—C23—H23A	109.5
C2—N1—O1	110.6 (3)	C24—C23—H23B	109.5
C15—N2—C16	114.7 (3)	H23A—C23—H23B	109.5
C22—N3—C21	116.2 (3)	C24—C23—H23C	109.5
C24—N4—O6	110.1 (3)	H23A—C23—H23C	109.5
C37—N5—C38	115.0 (3)	H23B—C23—H23C	109.5
C44—N6—C43	115.4 (3)	N4—C24—C25	125.5 (3)
C2—C1—H1A	109.5	N4—C24—C23	114.3 (3)
C2—C1—H1B	109.5	C25—C24—C23	120.1 (3)
H1A—C1—H1B	109.5	C24—C25—H25A	109.5
C2—C1—H1C	109.5	C24—C25—H25B	109.5
H1A—C1—H1C	109.5	H25A—C25—H25B	109.5
H1B—C1—H1C	109.5	C24—C25—H25C	109.5
N1—C2—C1	125.6 (3)	H25A—C25—H25C	109.5
N1—C2—C3	115.1 (3)	H25B—C25—H25C	109.5
C1—C2—C3	119.3 (3)	O6—C26—C27	111.3 (3)
C2—C3—H3A	109.5	O6—C26—H26A	109.4
C2—C3—H3B	109.5	C27—C26—H26A	109.4
H3A—C3—H3B	109.5	O6—C26—H26B	109.4
C2—C3—H3C	109.5	C27—C26—H26B	109.4
H3A—C3—H3C	109.5	H26A—C26—H26B	108.0
H3B—C3—H3C	109.5	O7—C27—C26	111.2 (3)
O1—C4—C5	111.9 (3)	O7—C27—H27A	109.4
O1—C4—H4A	109.2	C26—C27—H27A	109.4
C5—C4—H4A	109.2	O7—C27—H27B	109.4
O1—C4—H4B	109.2	C26—C27—H27B	109.4
C5—C4—H4B	109.2	H27A—C27—H27B	108.0
H4A—C4—H4B	107.9	O8—C28—O7	123.1 (3)
O2—C5—C4	110.3 (3)	O8—C28—C29	126.5 (3)
O2—C5—H5A	109.6	O7—C28—C29	110.4 (3)
C4—C5—H5A	109.6	O9—C29—C30	107.1 (3)
O2—C5—H5B	109.6	O9—C29—C28	109.2 (3)
C4—C5—H5B	109.6	C30—C29—C28	110.2 (3)
H5A—C5—H5B	108.1	O9—C29—H29	110.1
O3—C6—O2	123.1 (3)	C30—C29—H29	110.1
O3—C6—C7	126.2 (3)	C28—C29—H29	110.1
O2—C6—C7	110.7 (3)	C29—C30—H30A	109.5
O4—C7—C6	110.6 (3)	C29—C30—H30B	109.5
O4—C7—C8	106.0 (3)	H30A—C30—H30B	109.5
C6—C7—C8	109.6 (3)	C29—C30—H30C	109.5
O4—C7—H7	110.2	H30A—C30—H30C	109.5
C6—C7—H7	110.2	H30B—C30—H30C	109.5
C8—C7—H7	110.2	C32—C31—C36	120.0 (3)
C7—C8—H8A	109.5	C32—C31—O9	115.2 (3)
C7—C8—H8B	109.5	C36—C31—O9	124.8 (3)
H8A—C8—H8B	109.5	C31—C32—C33	121.0 (4)
C7—C8—H8C	109.5	C31—C32—H32	119.5
H8A—C8—H8C	109.5	C33—C32—H32	119.5
H8B—C8—H8C	109.5	C34—C33—C32	118.7 (3)
C14—C9—C10	120.8 (3)	C34—C33—H33	120.7
C14—C9—O4	124.3 (3)	C32—C33—H33	120.7
C10—C9—O4	115.0 (3)	C33—C34—C35	121.7 (3)
C9—C10—C11	119.8 (3)	C33—C34—O10	120.1 (3)
C9—C10—H10	120.1	C35—C34—O10	117.9 (3)
C11—C10—H10	120.1	C34—C35—C36	119.6 (4)
C12—C11—C10	119.0 (3)	C34—C35—H35	120.2
C12—C11—H11	120.5	C36—C35—H35	120.2
C10—C11—H11	120.5	C31—C36—C35	119.0 (4)
C11—C12—C13	122.3 (3)	C31—C36—H36	120.5
C11—C12—O5	120.6 (3)	C35—C36—H36	120.5
C13—C12—O5	116.7 (3)	N5—C37—O10	122.3 (3)
C12—C13—C14	119.2 (3)	N5—C37—C44	124.2 (3)
C12—C13—H13	120.4	O10—C37—C44	113.5 (3)
C14—C13—H13	120.4	N5—C38—C39	119.9 (3)
C9—C14—C13	118.9 (3)	N5—C38—C43	121.3 (3)
C9—C14—H14	120.5	C39—C38—C43	118.8 (3)
C13—C14—H14	120.5	C40—C39—C38	121.2 (3)
N2—C15—O5	122.2 (3)	C40—C39—H39	119.4
N2—C15—C22	124.8 (3)	C38—C39—H39	119.4
O5—C15—C22	113.0 (3)	C39—C40—C41	118.7 (3)
N2—C16—C17	119.3 (3)	C39—C40—H40	120.7
N2—C16—C21	121.3 (3)	C41—C40—H40	120.7
C17—C16—C21	119.4 (3)	C42—C41—C40	122.1 (3)
C18—C17—C16	121.0 (3)	C42—C41—Cl2	119.4 (3)
C18—C17—H17	119.5	C40—C41—Cl2	118.5 (3)
C16—C17—H17	119.5	C41—C42—C43	119.5 (3)
C17—C18—C19	118.7 (3)	C41—C42—H42	120.3
C17—C18—H18	120.6	C43—C42—H42	120.3
C19—C18—H18	120.6	N6—C43—C42	118.8 (3)
C20—C19—C18	122.1 (3)	N6—C43—C38	121.6 (3)
C20—C19—Cl1	119.8 (3)	C42—C43—C38	119.6 (3)
C18—C19—Cl1	118.1 (3)	N6—C44—C37	122.5 (3)
C19—C20—C21	119.4 (3)	N6—C44—H44	118.7
C19—C20—H20	120.3	C37—C44—H44	118.7
			
C4—O1—N1—C2	−170.3 (3)	O5—C15—C22—N3	176.7 (3)
C26—O6—N4—C24	−168.5 (3)	O6—N4—C24—C25	3.3 (5)
O1—N1—C2—C1	3.1 (5)	O6—N4—C24—C23	−178.5 (3)
O1—N1—C2—C3	−178.1 (3)	N4—O6—C26—C27	82.3 (3)
N1—O1—C4—C5	82.1 (3)	C28—O7—C27—C26	82.3 (3)
C6—O2—C5—C4	82.2 (3)	O6—C26—C27—O7	68.3 (4)
O1—C4—C5—O2	66.0 (4)	C27—O7—C28—O8	3.6 (4)
C5—O2—C6—O3	9.0 (4)	C27—O7—C28—C29	−175.0 (3)
C5—O2—C6—C7	−168.6 (3)	C31—O9—C29—C30	−169.9 (3)
C9—O4—C7—C6	68.8 (4)	C31—O9—C29—C28	70.8 (4)
C9—O4—C7—C8	−172.5 (3)	O8—C28—C29—O9	−1.5 (5)
O3—C6—C7—O4	9.4 (5)	O7—C28—C29—O9	177.0 (2)
O2—C6—C7—O4	−173.0 (3)	O8—C28—C29—C30	−118.8 (4)
O3—C6—C7—C8	−107.1 (4)	O7—C28—C29—C30	59.7 (4)
O2—C6—C7—C8	70.5 (4)	C29—O9—C31—C32	−173.2 (3)
C7—O4—C9—C14	13.2 (5)	C29—O9—C31—C36	6.1 (5)
C7—O4—C9—C10	−167.8 (3)	C36—C31—C32—C33	−0.5 (6)
C14—C9—C10—C11	−2.9 (5)	O9—C31—C32—C33	178.8 (3)
O4—C9—C10—C11	178.1 (3)	C31—C32—C33—C34	0.5 (6)
C9—C10—C11—C12	0.5 (5)	C32—C33—C34—C35	0.1 (6)
C10—C11—C12—C13	1.4 (6)	C32—C33—C34—O10	−173.6 (3)
C10—C11—C12—O5	−171.3 (3)	C37—O10—C34—C33	−88.6 (4)
C15—O5—C12—C11	−85.1 (4)	C37—O10—C34—C35	97.5 (4)
C15—O5—C12—C13	101.8 (4)	C33—C34—C35—C36	−0.6 (6)
C11—C12—C13—C14	−1.0 (6)	O10—C34—C35—C36	173.3 (3)
O5—C12—C13—C14	172.0 (3)	C32—C31—C36—C35	0.1 (6)
C10—C9—C14—C13	3.2 (5)	O9—C31—C36—C35	−179.2 (3)
O4—C9—C14—C13	−177.8 (3)	C34—C35—C36—C31	0.5 (6)
C12—C13—C14—C9	−1.3 (6)	C38—N5—C37—O10	−178.7 (3)
C16—N2—C15—O5	−178.5 (3)	C38—N5—C37—C44	0.6 (5)
C16—N2—C15—C22	0.9 (5)	C34—O10—C37—N5	4.8 (5)
C12—O5—C15—N2	9.3 (5)	C34—O10—C37—C44	−174.6 (3)
C12—O5—C15—C22	−170.1 (3)	C37—N5—C38—C39	−178.2 (3)
C15—N2—C16—C17	−177.4 (3)	C37—N5—C38—C43	0.6 (5)
C15—N2—C16—C21	1.4 (5)	N5—C38—C39—C40	179.1 (3)
N2—C16—C17—C18	179.6 (3)	C43—C38—C39—C40	0.3 (5)
C21—C16—C17—C18	0.8 (5)	C38—C39—C40—C41	1.7 (5)
C16—C17—C18—C19	1.0 (5)	C39—C40—C41—C42	−1.8 (5)
C17—C18—C19—C20	−0.8 (6)	C39—C40—C41—Cl2	178.5 (3)
C17—C18—C19—Cl1	178.9 (3)	C40—C41—C42—C43	−0.2 (5)
C18—C19—C20—C21	−1.2 (5)	Cl2—C41—C42—C43	179.5 (3)
Cl1—C19—C20—C21	179.2 (3)	C44—N6—C43—C42	180.0 (3)
C22—N3—C21—C16	0.5 (5)	C44—N6—C43—C38	−0.3 (5)
C22—N3—C21—C20	179.7 (3)	C41—C42—C43—N6	−178.0 (3)
N2—C16—C21—N3	−2.2 (5)	C41—C42—C43—C38	2.3 (5)
C17—C16—C21—N3	176.6 (3)	N5—C38—C43—N6	−0.8 (5)
N2—C16—C21—C20	178.5 (3)	C39—C38—C43—N6	178.0 (3)
C17—C16—C21—C20	−2.7 (5)	N5—C38—C43—C42	178.9 (3)
C19—C20—C21—N3	−176.5 (3)	C39—C38—C43—C42	−2.4 (5)
C19—C20—C21—C16	2.9 (5)	C43—N6—C44—C37	1.5 (5)
C21—N3—C22—C15	1.8 (5)	N5—C37—C44—N6	−1.8 (6)
N2—C15—C22—N3	−2.7 (6)	O10—C37—C44—N6	177.5 (3)

### Hydrogen-bond geometry (Å, º)

**Table:**

*D*—H···*A*	*D*—H	H···*A*	*D*···*A*	*D*—H···*A*
C4—H4*B*···O2^i^	0.99	2.60	3.337 (4)	131
C7—H7···O3^ii^	1.00	2.33	3.264 (4)	154
C14—H14···O3^ii^	0.95	2.47	3.389 (4)	164
C22—H22···O8^i^	0.95	2.53	3.468 (4)	170
C27—H27*A*···O5^ii^	0.99	2.56	3.284 (4)	130
C29—H29···O8^i^	1.00	2.33	3.173 (4)	142
C44—H44···N1^iii^	0.95	2.51	3.430 (5)	162
C10—H10···Cl2^iv^	0.95	2.91	3.855 (4)	174
C25—H25*A*···Cl2^v^	0.98	2.99	3.368 (4)	105
C32—H32···Cl1^vi^	0.95	2.89	3.748 (4)	151

Symmetry codes: (i) *x*−1, *y*, *z*; (ii) *x*+1, *y*, *z*; (iii) *x*+1, *y*, *z*−1; (iv) −*x*−1, *y*−1/2, −*z*; (v) *x*+1, *y*, *z*+1; (vi) −*x*+2, *y*+1/2, −*z*.
