# Supplementary figures and images for: Crystal structure of propaquizafop
Source: Acta Crystallogr Sect E Struct Rep Online. 2014 Nov 19;70(Pt 12):o1266–7. doi: 10.1107/S1600536814024751 (PMC4257450; doi:10.1107/S1600536814024751)

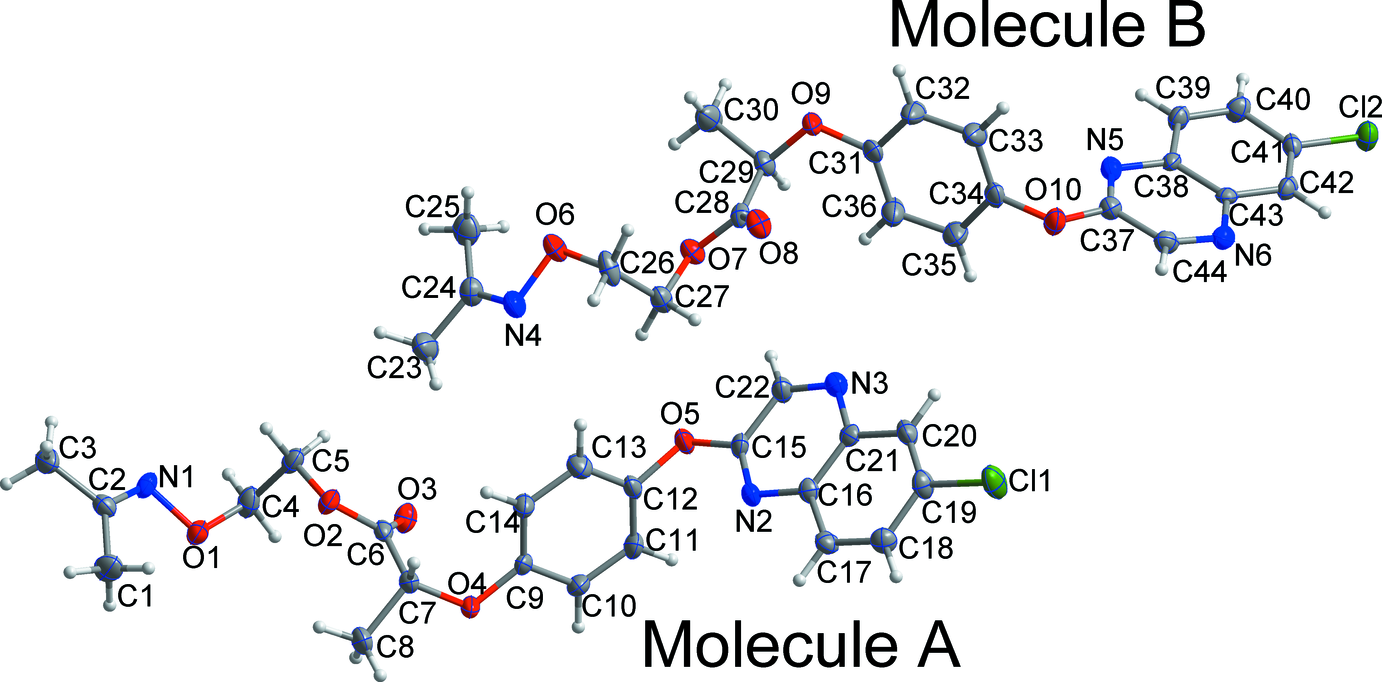

Supplement: Supplementary file 4 [file e-70-o1266-fig1.tif]

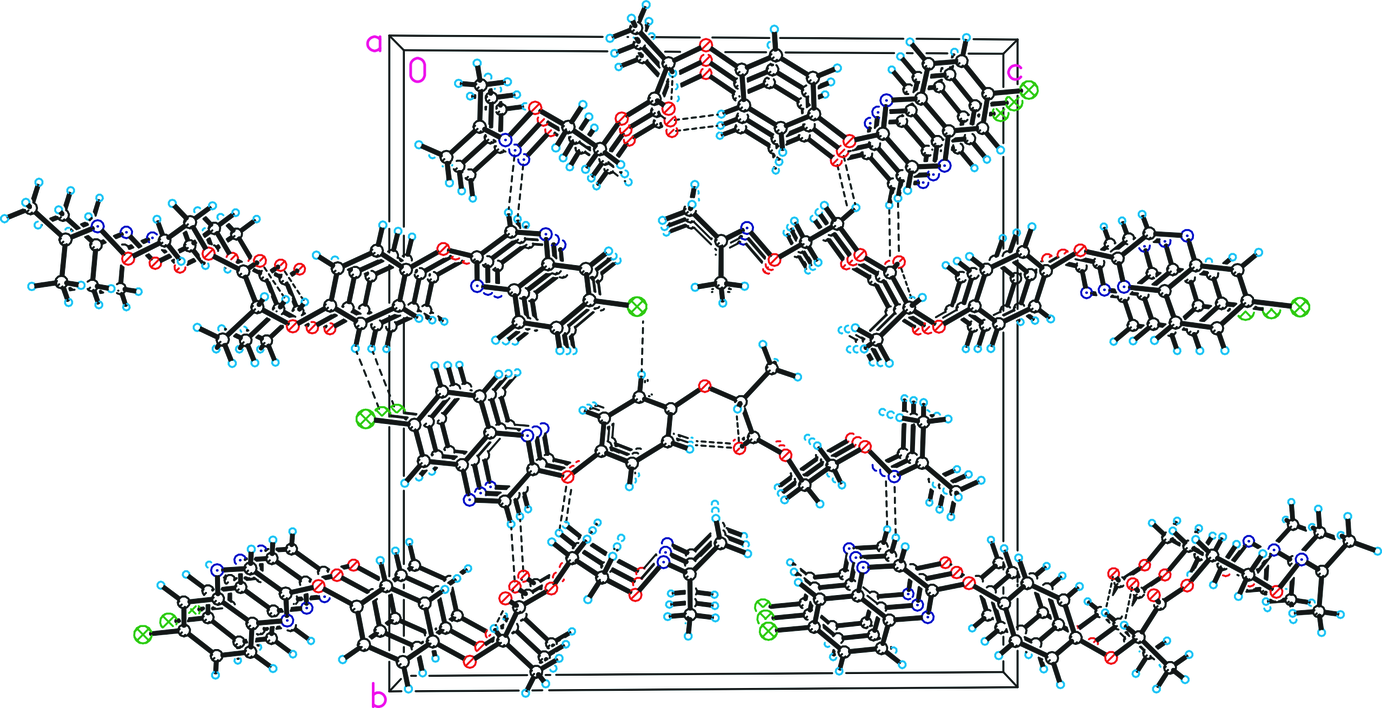

Supplement: Supplementary file 5 [file e-70-o1266-fig2.tif]
